# Study protocol: functioning curves and trajectories for children and adolescents with cerebral palsy in Brazil – PartiCipa Brazil

**DOI:** 10.1186/s12887-020-02279-3

**Published:** 2020-08-20

**Authors:** Paula S. C. Chagas, Carolyne M. Drumond, Aline M. Toledo, Ana Carolina de Campos, Ana Cristina R. Camargos, Egmar Longo, Hércules R. Leite, Kênnea M. A. Ayupe, Rafaela S. Moreira, Rosane L. S. Morais, Robert J. Palisano, Peter Rosenbaum, on Behalf of PartiCipa Brazil Research Group

**Affiliations:** 1 Graduate Program in Rehabilitation Sciences and Physical-Functional Performance, Universidade Federal de Juiz de Fora, Av. Eugênio do Nascimento, s / n - Dom Bosco, Juiz de Fora, Minas Gerais Brazil; 2 Graduate Program in Rehabilitation Sciences, Universidade de Brasília, Brasília, Brazil; 3 Graduate Program in Physical Therapy, Department of Physical Therapy, Universidade Federal de São Carlos, São Carlos, São Paulo, Brazil; 4 Graduate Program in Rehabilitation Sciences, School of Physical Education, Physical Therapy and Occupational Therapy, Universidade Federal de Minas Gerais, Belo Horizonte, Minas Gerais Brazil; 5 Graduate Program in Rehabilitation Sciences, Universidade Federal do Rio Grande do Norte, Faculdade de Ciencias da Saude do Trairi, Santa Cruz, Rio Grande do Norte Brazil; 6 Physiotherapy Course, Universidade de Brasília, Brasília, Brazil; 7 Department of Health Sciences, Universidade Federal de Santa Catarina, Araranguá, Santa Catarina Brazil; 8 Graduate Program in Health, Society and Environment and Department of Physiotherapy, Universidade Federal do Vale do Jequitinhonha e Mucuri, Diamantina, Minas Gerais Brazil; 9 College of Nursing and Health Professions, Drexel University, Philadelphia, USA; 10 McMaster University, CanChild Centre for Childhood Disability Research, Hamilton, ON Canada

**Keywords:** Cerebral palsy, International Classification of Functioning, Disability and Health - ICF, Participation, Activity, Gross motor function

## Abstract

**Background:**

Gross motor development curves for children with Cerebral Palsy (CP), grouped by Gross Motor Function Classification System (GMFCS) levels, help health care professionals and parents to understand children’s motor function prognosis. Although these curves are widely used in Brazil to guide clinical decision-making, they were developed with Canadian children with CP. Little is known about how these patterns evolve in children and adolescents with CP in low-income countries like Brazil. The PARTICIPA BRAZIL aims to: (i) to identify and draw a profile of functioning and disability of Brazilian children and adolescents with CP by classifying them, for descriptive purposes, with all five valid and reliable functional classifications systems (gross motor function, manual ability, communication function, visual and eating and drinking abilities); (ii) to create longitudinal trajectories capturing the mobility capacity of Brazilian children and adolescents with CP for each level of the GMFCS; (iii) to document longitudinal trajectories in the performance of activities and participation of Brazilian children and adolescents with CP across two functional classification systems: GMFCS and MACS (Manual Abilities Classification System); (iv) to document longitudinal trajectories of neuromusculoskeletal and movement-related functions and exercise tolerance functions of Brazilian children and adolescents with CP for each level of the GMFCS; and (v) to explore interrelationships among all ICF framework components and the five functional classification systems in Brazilian children and adolescents with CP.

**Methods:**

We propose a multi-center, longitudinal, prospective cohort study with 750 Brazilian children and adolescents with CP from across the country. Participants will be classified according to five functional classification systems. Contextual factors, activity and participation, and body functions will be evaluated longitudinally and prospectively for four years. Nonlinear mixed-effects models for each of the five GMFCS and MACS levels will be created using test scores over time to create prognosis curves. To explore the interrelationships among ICF components, a multiple linear regression will be performed.

**Discussion:**

The findings from this study will describe the level and nature of activities and levels of participation of children and youth with CP in Brazil. This will support evidence-based public policies to improve care to this population from childhood to adulthood, based on their prognosis.

## Background

Cerebral palsy (CP) refers to a group of developmental disorders of movement and posture due to a non-progressive impairment of the immature brain [1] that can affect health across all domains of functioning described by the International Classification of Functioning, Disability and Health (ICF) [2]. Evidence from developed countries shows that one in three children with CP does not walk, one in four does not speak, one in four has epilepsy, and one in 25 has hearing impairment [3].

Using *ICF concepts and language*, children with CP have primary impairments in *body structures and functions*, like muscle weakness and spasticity. Despite the non-progressive nature of the underlying brain damage, these impairments in the neuromusculoskeletal system and compensations due to the altered postural patterns may continue to progress [3, 4]. The association of dysfunctions and contextual factors usually results in *activity* limitations and *participation* restrictions that are secondary to the neurological impairments of this population [1, 2]. Regular assessments of functioning make it possible to chart progress and understand the evolution of the condition and the need to modify *contextual factors*, including therapeutic approaches, to achieve specific goals [5].

CP has traditionally been described in terms of clinical type, stratified into spastic unilateral (hemiplegia) or bilateral (diplegia and quadriplegia), dyskinetic or ataxic [3, 6, 7]. However, these descriptions do not describe what the child does from a functional point of view [8]. To address this reality, functional classifications have been developed, including the Gross Motor Function Classification System (GMFCS), Manual Ability Classification System (MACS), Communication Function Classification System (CFCS), Eating and Drinking Ability Classification System (EDACS) and Visual Function Classification System (VFCS) [8, 9]. It is important to highlight that functional classifications facilitate the exchange of consistent information among members of the interdisciplinary team and between the team and the family or the child/adolescent. In addition, the classifications standardize the population with CP for research purposes [8].

In 2002, Rosenbaum, Palisano and colleagues created the gross motor development curves for children with CP, based on 5-year longitudinal assessments of 657 Canadian children from across Ontario, reported according to the five levels of the GMFCS [10, 11]. These motor capacity curves help parents and healthcare professionals to understand patterns of motor development of children with CP, according to their functional level and age, as well as to predict their potential for motor acquisition and functional independence [11]. Targeting improved clinical applicability, centile reference curves based on the 66-item Gross Motor Function Measure (GMFM-66) were constructed by Hanna et al. (2008) [12]. These curves are widely used to guide clinical decision-making in Brazil, but all these tools were constructed based on the development of children with CP, aged 1 to 13 years, served by 19 publicly-funded children’s rehabilitation services in Ontario, Canada [11, 13]. Subsequently, Hanna et al. (2009) followed a sample of the study participants into adolescence and young adulthood [14]. Longitudinal trajectories and reference centiles were also developed in Canada and United States for several other outcomes, such as range of motion (Spinal Alignment and Range of Motion Measures - SAROMM), endurance (Early Activity Scale for Endurance - EASE), and strength (Functional Strength Assessment - FSA) [12] in young children with CP.

In the Netherlands, motor growth curves were created similar to those in Canada, despite differences in country, health service system and time period [15]. Trajectories for mobility, self-care [16] and participation [17] for Dutch individuals with CP across GMFCS levels were also developed. These studies have highlighted expected age-intervals at which motor and functional performance levels are achieved. Nevertheless, Van Gorp et al. (2018) observed that the development of motor performance in individuals with CP continues after gross motor capacity limits have been reached in childhood [18]. All the aforementioned studies addressed children and adolescents with CP from high-income countries.

Regarding the prevalence of functional levels, research has shown that the percentage of children with CP classified as ‘moderate to severe’ has decreased in Australia in the past decades [19]. In contrast, children with CP in low- and middle-income countries (LMIC) were reported to have more severe physical limitations and even higher rates of comorbidities compared to developed countries [20]. No previous studies have described the evolution of activity curves and participation trajectories of children and adolescents with CP in LMICs, such as Brazil – a country with diverse socioeconomic and cultural conditions that faces many challenges, such as access to public services and evidence-based rehabilitation treatments. The impact of these conditions on the development of children with disabilities is largely unknown.

A Brazilian population study is therefore needed to create activity curves and participation trajectories for children and adolescents with CP in Brazil, and to understand the relation of functional classification levels with body functions, activities and participation, across the life span. These curves would allow professionals to answer the following research questions: (1) Controlling for GMFCS levels, do Brazilian environmental factors influence the development of functioning of children and adolescents with CP? (2) What are the relationships among body functions, activities (capacity and performance) and participation across the life span in Brazilian children and adolescents with CP across functional classification levels? The specific research aims are:

(i) to identify and draw a profile of functioning and disability of Brazilian children and adolescents with CP by classifying them, for descriptive purposes, with all five functional classifications systems;
(ii) to create longitudinal trajectories capturing the mobility capacity of Brazilian children and adolescents with CP for each level of the GMFCS;
(iii) to document longitudinal trajectories in the performance of activities and participation of Brazilian children and adolescents with CP across the functional classification systems: GMFCS and MACS;
(iv) to document longitudinal trajectories of neuromusculoskeletal and movement-related and exercise tolerance functions of Brazilian children and adolescents with CP for each level of the GMFCS; and
(v) to explore interrelationship among all ICF framework components and the five functional classification systems in Brazilian children and adolescents with CP.

## Methods

### Design, participants and ethical approval

PARTICIPA BRAZIL will be a multicenter, longitudinal, prospective cohort study, in which Brazilian children and adolescents with CP (1 to 14 years of age) will be invited to participate, primarily at the Public Health System (*Sistema Único de Saúde* -SUS) and philanthropic services. Nine partners from seven Brazilian public universities have already agreed to participate in this study. These centers have hospitals or partnerships with public centers that collectively assist more than 500 children and adolescents with CP, mainly with physical therapy programs provided by trained professionals who are experienced in assessment and management of children with disabilities. The cities are located in strategic regions of Brazil – 4 universities/centers in the Southeast, 1 in the South, 1 the Middle Region of Brazil and 1 in the Northeast. Also, the project will be nationally advertised**.** Additional Brazilian public hospitals and public or philanthropic services will also be invited to participate. The assessments will be started only after the agreement of the parents, who will be asked to sign the Informed Consent Form. For children and adolescents, an assent form will be signed if the participant has the ability to do so. Ethical approval for this multicenter study was obtained before the start of the project at Federal University of Juiz de Fora (CAAE: 28540620.6.1001.5133).

#### Inclusion criteria

Children and adolescents diagnosed with CP, born after 2007, enrolled in rehabilitation services of Brazilian public university hospitals and partner services. Participants with clinical neuromotor characteristics and/or history consistent with CP, such as spasticity or mobility impairments, will be included in the study if, in the judgment of the health professionals providing their therapies, these children ‘look like’ they have CP, even if no formal diagnosis has been given, as it is often the case in Brazil.

#### Exclusion criteria

Children and adolescents with other recognized neuromotor dysfunctions, such as myelomeningocele, Down syndrome, or muscular dystrophies will be excluded from the study.

#### Control criteria

Children and adolescents with CP who have received botulinum toxin, selective dorsal rhizotomy, musculoskeletal or bone surgery, baclofen pump, or other technical interventions during the study period, will be included and followed, considering the time of these procedures as variables for separate analysis. All adaptive equipment and medications used by participants will be documented during the study follow-up. GMFM assessments will be done using the standard procedures, namely without the use of adaptive equipment. Note that although the use of these procedures and equipment will be documented, this study is not intended to investigate the specific effects of any of these interventions.

#### Sample size

Sample size calculations were performed using data from Scrutton and Rosenbaum [21]. Based on the GMFM-88 (Gross Motor Function Measure - 88 items version) and estimated score limits for a 10-year-old in each GMFCS stratum (98–100, 90–95, 60–80, 12–50 and < 10%, respectively), a sample of 150 children per GMFCS stratum would provide a power of 0.85 [11]. Based on the study by Palisano et al., a sample size of 700 children will be necessary for estimation of percentiles by age and GMFCS levels based on calculations for adequacy of the width of the 95% confidence interval (CI) for the 5th, 50th, and 95th percentiles [22, 23]. This sample size estimate is in accordance with other studies that investigated functional trajectories in children and adolescents in CP across the world [11, 15, 23–25].

### Instruments and procedures

**Fig. 1:**
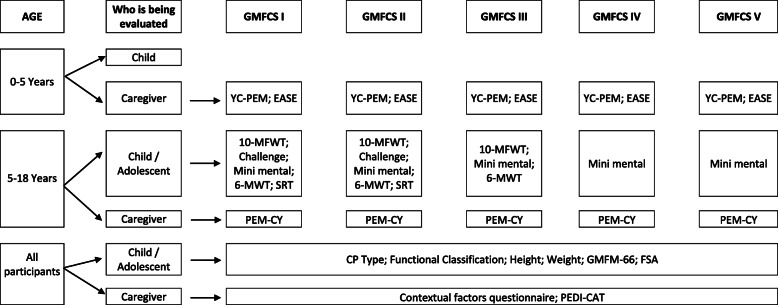
Flow-chart outlining the procedures of this longitudinal prospective cohort study after the inclusion of the child or adolescent in the study. Legend: GMFCS: Gross Motor Function Classification System; 10-MFWT: 10-Meter fast Walk Test; YC-PEM, Young Children’s Participation and Environment Measure; EASE: Early Activity Scale for Endurance; 6-MWT: 6-Minutes Walk Test; SRT: Shuttle Run Test; PEM-CY: Participation and Environment Measure for Children and Youth; CP: Cerebral Palsy; GMFM: Gross Motor Function Measure; FSA: Function Strength Assessment; PEDI CAT: Pediatric Evaluation of Disability Inventory-Computer Adaptive Test

All participating centers will perform data collection following the same procedures (Fig. 1). Table 1 shows the instruments that will be used to evaluate each component of the ICF, according to age and GMFCS level.

**Table 1:** Outcomes and assessment tools according to the International Classification of Functioning, Disability and Health - ICF

**Health condition**	CP clinical types: spastic (bilateral or unilateral), dyskinetic or ataxic. CP classifications: GMFCS, MACS, CFCS, EDACS, VFCS, FMS			
**Contextual factors**	**Outcomes**		**Assessment tools**	**Participants according to GMFCS or ages**
**Personal**	Name, age, gender, weight, height, educational level, life habits, history of other impairments, complaints and expectations			All
**Environmental**	Products and technology		For personal use: consumption (drugs), use in daily living (bath chair, orthotic devices), indoor and outdoor mobility and transportation (walking devices, wheelchairs, transfer devices), communication, culture, recreation and sports. Design, construction and building products and technology of buildings for public and private use, financial assets	All
	Support and relationships		Health professionals	
	Services, systems and policies		Transportation, social security and health services	
			Participation and Environment Measure for Children and Youth – PEM-CY	5 to 17 years
			Young Children’s Participation & Environment Measure - YC-PEM	0 to 5 years
**Functioning**	**Constructs**	**Domains**	**Assessment tools**	
**Activities and Participation**	Performance	^a^All 9 chapters	Participation and Environment Measure for Children and Youth – PEM-CY	5 to 17 years
			Young Children’s Participation & Environment Measure - YC-PEM	0 to 5 years
		^a^Chapters 5 to 9	Pediatric Evaluation of Disability Inventory Computer Adaptive Test – PEDI-CAT	0 to 18 years
	Capacity	Mobility	Gross Motor Function Measure – GMFM	GMFCS I to V
			10 Meter Fast Walk Test - 10mFWT	GMFCS I to III (5 to 18 years)
			Gross Motor Function Measure - Challenge Module	GMFCS I to II (5 to 18 years)
**Body Functions**	Mental functions		Mini mental State Examination - MMSE	5 to 14 years
	Exercise tolerance functions		Early Activity Scale for Endurance - EASE	18 months to 5 years
			Six Minute Walk Test – 6mWT	GMFCS I to III (5 to 18 years)
			Shuttle Run Test - SRT	GMFCS I e II (7 to 18 years)
	Muscle power functions		Functional Strength Assessment - FSA	> 18 months

Legend: *CP* cerebral palsy, *GMFCS* Gross Motor Function Classification System, *MACS* Manual Ability Classification System, *CFCS* Communication Function Classification System, *EDACS* Eating and Drinking Ability Classification System, *VFCS* Visual Function Classification System, *FMS* Functional Mobility Scale; ^a^ chapters of activities and participation part of International Classification of Functioning, Disability and Health

Children < 6 years will be evaluated every 6 months, and the children and adolescents ≥6 years of age will be evaluated annually. We expect to have at least one evaluation per year, during the 4 years follow-up, totaling a minimum of four evaluations per participant. For the constructions of the curves, it is essential to have at least three longitudinal evaluations per child [26]. The examiners, mainly physical and occupational therapists, will receive pre-study training, both on the theory and practical applications for all instruments and classifications. Examiners should have agreement above 80% (intra-class correlation coefficient = ICC ≥ 0.80) against criterion tests – to be assessed during training and every year during the study procedures – to check their reliability.

Some measures will be performed with the child or adolescent and another few with the caregiver (as can be seen in Fig. 1). Also, the number of measures that will be applied will depend on the functional classification of the participant (the more functional ones will receive more assessments, but also the more functional the participant, the faster the tests will be completed). There will be different assessors for the child/adolescent and for the parents. The time spent in each measurement will depend on the motor ability of the child, but we estimate a mean time of 90 minutes of evaluation. Participants will take breaks if needed. As the evaluation will occur once a year or twice yearly (in children under 6 years old), it may be necessary to split the tests into two visits (maximum of 1 week apart) to avoid burdening up the participant.

For descriptive purposes, the children will be described according to clinical type, such as spastic unilateral or bilateral, dyskinetic or ataxic and according to their functional classification. The main caregiver will complete a questionnaire about the contextual factors of the participant, including: personal factors (e.g., health status, age, gender, educational level, life habits, history of other impairments) and environmental factors (e.g., orthotic devices, wheelchairs, transfer devices, access to health services), as described in Table 1. The family’s economic level will be accessed by means of the Brazilian Economic Classification Criteria (BECC) [27].

The participants will also have their weight and height measured using standardized instruments. Weight will be measured on a digital scale calibrated to zero, in Kilograms, with the child undressed or by taking the difference between the weight of the parent with and without the child on their lap. Height will be measured in centimeters by stadiometer, in the supine or standing position, in those children who do not have significant musculoskeletal deformities (e.g., scoliosis, kyphosis or flexion deformities of the lower limbs). In children who present deformities, height will be estimated by the length between the knee and the heel (anterior surface of the leg to the sole of the foot), using a stadiometer, applying the formula of Stevenson (1995), where: height = (2.69 x knee length) + 24.2 [28].

### Activity and participation measures

#### Functional Classifications Systems

Participants’ mobility will be classified by the valid and reliable GMFCS [10]. GMFCS uses a five-level ordinal scale to describe the level of independence in postural control and mobility of children and adolescents with CP [10, 29], stratified by age bands: < 2 years, 2 to < 4 years, 4 to < 6 years, 6 to < 12 years, and 12 to 18 years of age. Level I describes the most functional children, who walk independently and go up and down stairs without assistance, whereas level V represents children with the least function, being fulltime wheelchair users, with limited head and trunk control [10, 29].

The MACS, the CFCS, the EDACS and VFCS – analogues of the GMFCS with good validity and reliability – will be also used to document the functioning of the children and adolescents across five levels, in the same way of the GMFCS [8, 9, 14, 30, 31]. All the functional classifications have five levels, where level I represents the most independent children or adolescents, and level V represents children or adolescents who require most assistance in the respective functional domain. All of these measures are standardized, reliable, valid and complementary to one another [8, 9, 14, 31]. The classification levels of each of these instruments at childhood are summarized in Table 2.

**Table 2:** Five classification levels of the Gross Motor Functional Classification System (GMFCS), the Manual Ability Classification System (MACS), the Communication Function Classification System (CFCS), the Eating and Drinking Ability Classification System (EDACS) and Visual Function Classification System (VFCS)

Levels	GMFCS	MACS	CFCS	EDACS	VFCS
I	Walk without limitation	Handles objects easily and successfully	Sends and receives efficiently with others	Eats and drinks efficiently	Use visual function with successfully
II	Walk with limitations	Handles objects but with reduced quality and/or speed of achievement	Sends and receives with others but may need extra time	Eats and drinks safely but with some limitations to efficiency	Uses visual function successfully but needs compensatory strategies
III	Walk using a hand-held mobility device	Handles objects with difficulty; needs help to prepare and/or modify activities	Sends and receives with familiar partners effectively, but not with unfamiliar partners	Eats and drinks but may be limitations to efficiency	Uses visual function but needs some adaptations
IV	Self-mobility with limitations; may use powered mobility	Handles a limited selection of easily managed objects in adapted situations	Inconsistently sends and/or receives even with familiar partners	Eats and drinks with significant limitations to safety	Need very adapted environments but performs just part of vision-related activities
V	Transported in manual wheelchair	Does not handle objects and has mostly limited ability to perform actions	Seldom effectively sends and receives, even with familiar partners	Unable to eat and drink safely – tube feeding may be considered	Does not use visual function even in very adapted environments

Legend: *GMFCS* Gross Motor Function Classification System, *MACS* Manual Ability Classification System, *CFCS* Communication Function Classification System, *EDACS* Eating and Drinking Ability Classification System, *VFCS* Visual Function Classification System

The mobility performance of the children aged 4–18 years in home, school and community will be classified using the Functional Mobility Scale (FMS) [32, 33]. The FMS rates walking ability at three specific metric distances based on three environments: 5 m (home), 50 m (school) and 500 m (community). Opposite to the GMFCS ratings, in FMS children in level 1 use wheelchairs and children in level 6 are independent on all surfaces [32, 33]. The participants will be classified by trained therapists in the first assessment and reclassified in subsequent assessments for all classification systems.

To assess children’s gross motor capacity, we will use the following tools: Gross Motor Function Measure (GMFM-66) [34], the Gross Motor Function Measure-Challenge Module [35], and 10-m fast walk test [36]. The GMFM-66 is a quantitative clinical tool that assesses gross motor activity with the purpose of measuring changes in children with CP over time [34]. The items are grouped into five dimensions: A: lying and rolling; B: sitting; C: crawling and kneeling; D: standing; E: walking, running and jumping. Items are scored on a four-point ordinal scale (specifically defined for each of the four scores for every item): 0 = does not perform; 1 = starts an activity; 2 = partially completes the activity; 3 = complete the activity as described in the GMFM-66 manual. In this study, we will compute the Rasch analysis-based GMFM-66 scores, providing an interval scale using the new GMAE-3 application (Gross Motor Ability Estimator – 3rd version) [34].

The Challenge Module, composed of 28 items, measures more complex gross motor activities. It was created for children and adolescents with CP in GMFCS levels I and II (if GMFCS II, minimum GMFM-66 score arbitrarily set at 70 to reflect the higher end of the Level II ability spectrum) [11], 5 to 18 years of age, able to follow instructions for a motor skill test. The test includes 17 locomotor items and 7 object control items. The mean score of three trials is calculated for each item and the total of these means reported. Scores ranged from 0 to 112 [37].

Walking capacity will be evaluated by the 10-m fast walk test (10mFWT) for children from 4 to 18 years of age [36, 38]. The 10mFWT has the potential to provide valuable clinical information regarding gait abilities and outcomes in ambulatory children (GMFCS I, II and III) able to walk 10 m with or without a walking aid [36, 38]. It is safe, easy, inexpensive to administer and allows us to calculate the walking speed for the minimum distance required for functional ambulation.

To assess children’s performance in activities and participation the following tools will be used: Pediatric Evaluation of Disability Inventory – Computer Adaptive-test (PEDI-CAT) [39], Young Children’s Participation & Environment Measure (YC-PEM), and Participation & Environment Measure for Children and Youth (PEM-CY) [40, 41]. The PEDI-CAT was developed to measure performance in daily activities, mobility, cognitive-social, and responsibility in children and adolescents up to 21 years of age [39]. The application requires a computer with the instrument software installed and can be self-administered (i.e., completed by the child’s parents) or through a parent interview with a professional [39, 42]. In the domains of daily activities, mobility, and cognitive social, the four-point scores are based on different levels of difficulty. The responsibility domain classifies items on a five-point scale, describing the sharing of responsibility between caregiver and child or adolescent in managing complex, multi-step life tasks. The overall score is transformed to a normative score (based on age) and a continuous score that will be used in the analyses. The PEDI-CAT has been translated and adapted culturally to Brazil [43].

The YC-PEM and the PEM-CY are parent-completed measures that look at participation of children and youth, aged 0–5 years and 5–17 years, respectively, in the home, daycare/preschool (YC-PEM) or school (PEM-CY) and community [40, 41]. Both instruments capture parent/caregiver perspectives of the child’s frequency of attending activities, level of involvement (i.e., engagement in the activities) and satisfaction with valued activities, and of the supports, barriers, resources and helpfulness of the environment in those 3 settings. Both instruments (YC-PEM and PEM-CY) have been translated and adapted culturally to Brazil [44, 45]. In this study, we will analyze: 1) frequency of attendance (rated using an eight-point scale with response options varying from daily to never); 2) level of involvement (five-point scale with response from minimally to very involved); and 3) change desired (yes or no). Activities in a setting are summed to provide a frequency score per setting. Environment scores (percentages) will be used in the description of contextual factors and their relationships with other ICF components.

#### Body functions measures

Neuromusculoskeletal and movement-related functions will be evaluated by Functional Strength Assessment (FSA) [46]. The FSA provides an estimate of strength for major muscle groups including the neck and trunk flexors and extensors, hip extensors, knee extensors and shoulder flexors bilaterally [46]. Items are scored on a 5-point ordinal scale of 1 (only flicker of contraction or just initiates movement against gravity) to 5 (full available range against gravity and strong resistance) [46].

Exercise tolerance will be measured by: Early Activity Scale for Endurance (EASE) [47], Six Minute Walk Test (6MWT) [48, 49] and Shuttle Run Test (SRT) [50, 51]. The EASE is a parent-completed questionnaire of the child’s perceived endurance for activity in young children with cerebral palsy, until 5 years old, including frequency, intensity, duration, and type of physical activity [47]. Items are scored on a 5-point ordinal scale from 1 = Never to 5 = Always, with higher scores indicating greater exercise tolerance [47]. The 6MWT is a submaximal test that assesses the tolerance for walking a prolonged distance with or without walking aid in children and adolescents from 4 to 18 years of age in GMFCS levels I, II and III. The greater the distance covered in six minutes the better the exercise tolerance [48, 49]. In the SRT participants will walk or run between 2 markers delineating the respective course of 10 m at a set incremental speed determined by a signal (every minute) [50, 51]. The starting speeds for the tests are 5 and 2 km/h for participants who are classified at GMFCS I and II, respectively, and the speeds are increased by 0.25 km/h every minute [50, 51]. The last completed level (accurate to a half shuttle) will be recorded and used for analysis. This test has been shown to be reliable, valid, and sensitive to change in children with CP [50, 51].

Mental functions will be screened by Mini-Mental State Examination (MMSE) adapted for children and translated to Portuguese-Brazil [52, 53]. The MMSE evaluates and monitors five areas of cognitive function: orientation, attention/concentration, registration, recall and language [52]. The score ranges from 0 to 37 points and age-group cut-off abnormal values were established for: 3–5 years (24 points); 6–8 years (28 points); 9–11 years (30 points); 12–14 years (35 points) [52].

### Statistical analysis

Initially, the data will be explored descriptively, and the assumptions of normality will be tested using the Kolmogorov-Smirnov test. Moreover, Q-Q plots will be used to verify which distribution best fits the data.

To identify and draw a profile of functioning and disability, categorical variables will be presented through frequencies (and percentages) and numerical variables through means and standard deviations, using all five functional classification systems.

Longitudinal trajectories will be created describing the average change in gross motor function, activity and participation, between different ages, using nonlinear mixed-effects models fit for each of the five GMFCS and MACS levels. For the mobility capacity trajectory curves, the GMFCS will be used; for activities and participation trajectory curves, we will use the GMFCS and MACS; and for neuromusculoskeletal, movement-related and exercise tolerance functions, the GMFCS will be used. Random effects (e.g., age) will be fitted for each parameter to estimate the variability in the true change parameters among children.

Each model will consider two parameters: rate and limit of development (average maximal performance level for a subgroup). To enhance interpretation, the rate parameters will be used to calculate the average age by which individuals will reach 90% of the limit (age-90). The 95% CI of the limit and age-90 will be calculated and used to detect differences between GMFCS and MACS levels.

We will adopt a Multiple Regression analysis to explore the interrelationship between each of the functional classifications (GMFCS, MACS, CFCS, EDACS and VFCS) as a response variable, and the predictor variables (personal and environmental factors, capacity, performance and functional outcomes). Stepwise, starting from linear to cubic fitting regressions, will be used to generate equations for predicting personal and environmental factors, capacity, performance, and functional outcomes. To avoid collinearity, Spearman’s test will be applied to correlate all the predictor variables. The correlation matrix will be analysed, and variables that to exhibit a high correlation will be considered collinear. Correlation coefficient values will be classified as very weak (below 0.20); weak (0.20 to 0.39); moderate (0.40 to 0.69); high (0.70 to 0.90) and very high (> 0.90) [54]. Personal and environmental factors presenting categorical variables will be considered as dummy variables.

All data collected in the different centers will be inserted in a password-secured identified Excel spreadsheet. Statistical analysis will be performed in Statistical Package for Social Sciences (SPSS©, version 25).

## Discussion

### Application of study results

It is expected that through this study, Brazilian therapists will be able to apply longitudinal trajectories validated for Brazilian children, serving as a guide for clinical decision-making. In addition, the findings from this study are expected to help us to describe and understand activities and participation, neuromusculoskeletal and movement-related functions and exercise tolerance functions of children and youth with CP in Brazil across the spectrum of functional levels and the different geographical regions of Brazil. This will make it possible to propose evidence-based public policies to improve services to this population in different stages of life, from childhood to adulthood, according to their motor prognosis and phase of motor evolution.

Being able to report levels of activities and participation will support the arguments for higher and most appropriate investments in treatment and assistive technologies during important phases of these children’s lives. This should help to promote their best capacity and quality of life to improve their participation in society and that of their families. Finally, it is expected that this study will inform us about the relationships among the different domains of the ICF and its contextual factors in Brazilian children and adolescents with CP. These findings will allow therapists to better understand important factors that influence their clinical decisions, and potentially expand the range of services and advice they have to offer.

### Potential risks and challenges

We may experience some difficulties in the follow-up of the children and youth across the years. To try to control this problem we will explain to the caregivers the importance of the study to their child and to the understanding of the care needed for children with CP in Brazil. We will also build in a number of tracking strategies for the children and families, including sending the children birthday cards, sending families annual study newsletters and asking each family at the start of the study for a contact (e.g., grandparents) who could help us find families that move to another house during the study. One another strategy to maintain the families in the study is that we will give a report after each evaluation, with broad treatment guidelines and ideas for adaptive equipment and technologies that might be useful.

### Dissemination of results

We plan to participate in conferences, to present the project and the results in plain language to all family participants (caregivers) and children and youth, and to CP organizations and services. We will disseminate the results of the study in papers in high impact peer-reviewed journals. All knowledge translation activities will be done in both Brazilian Portuguese and English. This study will permit the development of strategies of knowledge translation to Brazilian citizens, to illustrate that children and youth with CP have different prognoses according to their functional level, and that they can participate and be integrated in daily life activities and leisure during their childhood regardless of functional level.

### Future research

The results of this study may help professionals to advocate for the development of future research regarding the access of Brazilian children and adolescents with CP to appropriate equipment and orthoses; to investigate the effects of interventions focusing on providing enrichment of activities and participation; and to inform public policies towards better access to health services considering the variability of the contextual factors across the country. Also, we believe that after the development of this study, studies that investigate the knowledge and implementation of the ‘F-words for Childhood Development’ in low-income countries, like Brazil, will have created a big difference in the profile of the families [55, 56]. Our PartiCipa Brazil Team advocates for these studies.

## Data Availability

not applied at this moment.

## Abbreviations

10mFWT: 10-m fast walk test
6MWT: Six Minute Walk Test
BECC: Brazilian Economic Classification Criteria
CFCS: Communication Function Classification System
CP: Cerebral Palsy
EASE: Early Activity Scale for Endurance and strength
EDACS: Eating and Drinking Ability Classification System
FMS: Functional Mobility Scale
FSA: Functional Strength Assessment
GMAE-3: Gross Motor Ability Estimator – 3rd version
GMFCS: Gross Motor Function Classification System
GMFM-66: Gross Motor Function Measure (66 items version)
GMFM-88: Gross Motor Function Measure (88 items version)
ICC: Intra-class correlation coefficient
ICF: International Classification of Functionality, Disability and Health
LMIC: Low- and middle-income countries
MACS: Manual Ability Classification System
MMSE: Mini-Mental State Examination
PEDI-CAT: Pediatric Evaluation of Disability Inventory – Computer Adaptive-test
PEM-CY: Participation & Environment Measure for Children and Youth
ROM: Range of motion
SAROMM: Spinal Alignment and Range of Motion Measures
SPSS: Statistical Package for Social Sciences
SRT: Shuttle Run Test
SUS: *Sistema Único de Saúde*
VFCS: Visual Function Classification System
YC-PEM: Young Children’s Participation & Environment Measure
